# Maize Domestication and Anti-Herbivore Defences: Leaf-Specific Dynamics during Early Ontogeny of Maize and Its Wild Ancestors

**DOI:** 10.1371/journal.pone.0135722

**Published:** 2015-08-12

**Authors:** Daniel Maag, Matthias Erb, Julio S. Bernal, Jean-Luc Wolfender, Ted C. J. Turlings, Gaétan Glauser

**Affiliations:** 1 Laboratory of Fundamental and Applied Research in Chemical Ecology, University of Neuchâtel, Rue Emile-Argand 11, 2000, Neuchâtel, Switzerland; 2 Laboratory of Phytochemistry and Bioactive Natural Products, School of Pharmaceutical Sciences, University of Geneva, University of Lausanne, Quai Ernest-Ansermet 30, 1211, Geneva, Switzerland; 3 Institute of Plant Sciences, University of Bern, Altenbergrain 21, 3013, Bern, Switzerland; 4 Department of Entomology, Texas A&M University, College Station, TX, 77843–2475, United States of America; 5 Neuchâtel Platform of Analytical Chemistry, University of Neuchâtel, Avenue de Bellevaux 51, 2000, Neuchâtel, Switzerland; Texas A&M University, UNITED STATES

## Abstract

As a consequence of artificial selection for specific traits, crop plants underwent considerable genotypic and phenotypic changes during the process of domestication. These changes may have led to reduced resistance in the cultivated plant due to shifts in resource allocation from defensive traits to increased growth rates and yield. Modern maize (*Zea mays* ssp. *mays*) was domesticated from its ancestor Balsas teosinte (*Z*. *mays* ssp. *parviglumis*) approximately 9000 years ago. Although maize displays a high genetic overlap with its direct ancestor and other annual teosintes, several studies show that maize and its ancestors differ in their resistance phenotypes with teosintes being less susceptible to herbivore damage. However, the underlying mechanisms are poorly understood. Here we addressed the question to what extent maize domestication has affected two crucial chemical and one physical defence traits and whether differences in their expression may explain the differences in herbivore resistance levels. The ontogenetic trajectories of 1,4-benzoxazin-3-ones, maysin and leaf toughness were monitored for different leaf types across several maize cultivars and teosinte accessions during early vegetative growth stages. We found significant quantitative and qualitative differences in 1,4-benzoxazin-3-one accumulation in an initial pairwise comparison, but we did not find consistent differences between wild and cultivated genotypes during a more thorough examination employing several cultivars/accessions. Yet, 1,4-benzoxazin-3-one levels tended to decline more rapidly with plant age in the modern maize cultivars. Foliar maysin levels and leaf toughness increased with plant age in a leaf-specific manner, but were also unaffected by domestication. Based on our findings we suggest that defence traits other than the ones that were investigated are responsible for the observed differences in herbivore resistance between teosinte and maize. Furthermore, our results indicate that single pairwise comparisons may lead to false conclusions regarding the effects of domestication on defensive and possibly other traits.

## Introduction

The term domestication refers to the process of genetic modification of wild species *via* artificial selection leading to cultivars that are adapted to human needs [1]. Prevailing theory supposes that modern crop varieties have lost, at least, part of their herbivore resistance during the course of domestication and are therefore more susceptible to herbivorous insects [2, 3], although the impact of cultivation on plant defence may vary with different domestication events [4]. Three underlying mechanisms have been proposed to explain such enhanced susceptibility [4]. Firstly, certain plants may have been selected for increased nutritive quality thereby also increasing the performance and fitness of herbivores [5]. Secondly, selective breeding may have favoured a reduction in defensive secondary metabolites to reduce toxicity and enhance palatability, e.g. selection of low glucosinolate-containing cultivars in the genus *Brassica* [6]. And lastly, according to the resource allocation hypothesis, selection for increased plant growth and yield may have resulted in a concomitant reduction of plant defences as the result of a trade-off between the two traits [7, 8].

The domestication of maize (*Zea mays* ssp. *mays*) took its beginning in a single event in the south of present-day Mexico approximately 9000 years ago. After persistent controversy in the scientific community the wild ancestor of maize was finally identified as Balsas teosinte (*Zea mays* ssp. *parviglumis*), a grass whose natural habitat is the Balsas River watershed [9–12]. The term teosinte is collectively applied to all taxa within the genus *Zea* except for maize and there is evidence that several of these taxa are able to hybridise with the latter [13–16]. Maize and Balsas teosinte differ remarkably in their phenotypic appearance, yet, only about 1200 genes (corresponding to 2–4% of the maize genome) were targeted during human selection [17]. Furthermore, domestication only imposed modest effects on the genetic diversity of maize: it has been estimated that about 80% of the wild ancestor’s genetic variability has been preserved [14]. Traces of natural hybridisation between maize and the more distant annual teosinte, *Z*. *mays* ssp. *mexicana*, can also be detected in the maize genome. Up to 20% of genetic admixture from *Z*. *mays* ssp. *mexicana* has been found in Mexican maize varieties depending on the altitude at which the crop is grown [16].

Deliberate introgression of teosinte-maize hybrids into maize crops has been reported as a common practice among Mexican farmers in order to improve the crop’s germplasm [18]. This coincides with evidence that teosinte plants are more resistant against herbivory than cultivated maize varieties [13]. Almost two decades ago Rosenthal and Dirzo [19] presented support for the resource allocation model in the teosinte/maize system: whereas growth rates and yield increased along a domestication gradient and were highest for a modern hybrid line, herbivore resistance declined along the same gradient. Accordingly, perennial and annual teosintes were found to experience significantly less damage from herbivores than maize both under semi-field conditions and in the laboratory. In a more recent study, plants of Balsas teosinte growing as weed within Mexican maize fields also displayed lower injury rates from fall armyworm (*Spodoptera frugiperda*) infestation than neighbouring maize plants during three subsequent years [18]. Furthermore, teosintes and maize plants have been shown to differ in their expression profiles of four defence-related genes following *S*. *frugiperda* infestation [20]. Elevated expression levels of these genes, in particular two protease inhibitors, in teosinte correlated with decreased caterpillar growth and development. Similarly, domestication has affected the suitability of the plant for another specialist herbivore, the corn leafhopper (*Dalbulus maidis*). Female adults were found to preferentially lay their eggs on maize compared to Balsas teosinte [21] and the performance of their offspring increased with the domestication status of the host plant [22]. This gradient in performance was associated with an inverted gradient of leaf toughness [21].

Besides physical defences or the expression of protease inhibitors most plants also possess a variety of toxic or repulsive defence metabolites [23]. In many grasses, including young maize seedlings, 1,4-benzoxazin-3-ones (BXs) are the predominant class of defensive secondary metabolites [24]. BXs are stored as inactive 2-*O*-*β*-D-glucosides in the vacuole. Following tissue disruption, e.g. during herbivory, they come into contact with β-glucosidases, which are stored separately in the plastids. These enzymes hydrolyse the glycosidic bond thereby releasing the active BX aglucone [25, 26]. BXs mediate resistance against a broad range of herbivores and pathogens in maize [24], e.g. the European corn borer (*Ostrinia nubilalis*) [27] or *Setosphaeria turcica*, the causal agent of northern leaf blight [28]. However, certain herbivore species have adapted to this line of defence. While different *Spodoptera* spp. are able to reglucosylate, and thus detoxify selected BX aglucones [29–32], larvae of the western corn rootworm (*Diabrotica virgifera virgifera*) even possess complete tolerance to high BX levels [33, 34].

In addition to BXs, maize plants possess polyphenolic compounds with feeding-deterrent and toxic properties. One of the best studied of these compounds is maysin [2”-*O*-α-L-rhamnosyl-6-*C*-(6-deoxy-*xylo*-hexos-4-ulosyl)-luteolin], a C-glycoslyated flavone [35]. Originally isolated from maize silks, maysin has been shown to negatively affect the growth of corn earworm (*Helicoverpa zea*) caterpillars as well as that of larvae of the fall armyworm (*S*. *frugiperda*), both in laboratory bioassays as well as in the field [36, 37]. Furthermore, genes that are involved in maysin synthesis have been related to resistance against different lepidopteran pests [38–40].

It is known that defensive traits vary with plant age [41] and the resulting quantitative and/or qualitative changes have been subject to several studies, but contrasting patterns have been found. Glucosinolate levels, for instance, increase with plant age in *Brassica oleracea* [42] whereas their contents decrease during early vegetative growth in *Brassica juncea* [43]. With regard to maize, it has been reported that BX concentrations are highest in young seedlings and decrease in a leaf-specific manner during early ontogeny [44, 45]. By contrast, leaf toughness has been found to increase with plant age [46]. To the best of our knowledge nothing is known yet about the ontogenetic trajectories of defence traits in the wild ancestor of maize. Here we compared age-related changes of leaf toughness and the levels of two classes of secondary metabolites, N-containing BXs and the glycosylated flavonoid maysin, for teosinte and cultivated maize varieties during early vegetative growth. In order to account for differential effects of leaf ontogeny and plant age, all traits were measured in different leaves at each growth stage. Based on the existing literature we predicted that (I) leaf toughness increases with plant age, but is unaffected by leaf ontogeny, (II) levels of secondary defence metabolites are highest in young seedlings and decrease thereafter irrespective of domestication status, and (III) defence traits are expressed more strongly in teosinte compared to cultivated maize.

## Materials and Methods

### Plant material & growing conditions

Seeds of the two teosinte accessions T62 (PI384062; *Zea mays* ssp. *parviglumis*) and T77 (PI566677; *Zea mays* ssp. *mexicana*) were obtained from the USDA National Plant Germplasm System. Accession T62 was originally collected 1km south of the town of Palo Blanco in Guerrero state, Mexico (17°25’N, 99°30’W) and T77 was originally collected near the town of Penjamillo in Michoacan state, Mexico (20°10’N, 101°52’W). Seeds of the teosinte population Ejutla were collected in Jalisco State, Mexico (Ejutla municipality, 19°54’N, 104°10’W; [22]) whereas the Tuxpeño landrace maize seeds (*Z*. *mays* ssp. *mays*) were collected from two different locations: Talpitita (Villa Purificaciòn municipality, 19°43’N, 104°48’W) and El Cuyotomate (Ejutla municipality, 19°58’N, 104°04’W). Seeds of both the teosinte population from Ejutla and the Tuxpeño landrace were collected on private land with permission of the respective owners. Seeds of the maize varieties Delprim and B73 were obtained from Delley semences et plantes (Delley, Switzerland) and those of the maize variety Pactol were obtained from Novartis (St. Sauveur, France). The three maize varieties originate from temperate zone breeding programs and are known to differ in inducible chemistry [47–49].

### Growing conditions & sample collection

For the first experiment, plants of the teosinte population Ejutla and of the maize cultivar B73 were grown in commercial soil (Aussaaterde, Ricoter Erdaufbereitung AG, Aarberg, Switzerland) in bottom-pierced plastic pots (Ø 4 cm, 11 cm high). Between growth stages L4 and L5 (‘leaf-over’ ranking, OMAFRA Publication 75, Guide to Weed Control, 2014–2015, [45]) plants were transferred to larger pots (Ø 12 cm, 18 cm high). All plants were grown in a completely randomised manner under natural light conditions in a greenhouse at the University of Neuchâtel (Switzerland) during late June and July 2012 and watered as needed.

Plants for the second experiment were grown during May and early June 2014 under the same conditions as above. Plants of the teosinte accessions T62 and T77, of the Tuxpeño landrace (Talpitita and El Cuyotomate populations) and of the maize varieties Pactol and Delprim were directly grown in large plastic pots (Ø 11.7 cm, 13.5 cm high) in a fully randomised design.

To determine the temporal dynamics of secondary metabolite accumulation and leaf toughness during early vegetative growth stages in the different plant genotypes individual leaves were harvested at three growth stages, i.e. L2, L4 and L6. For each growth stage the second leaf and the youngest almost fully expanded leaf, which was already arching over, were excised. Leaves were cut at their base and divided into halves along the midrib. One half was immediately snap-frozen in liquid nitrogen and stored at -80°C until extraction of defence metabolites. The other half (carrying the midrib) was used to measure leaf toughness. During the first experiment leaves were excised and directly snap-frozen in liquid nitrogen without being cut into halves as only chemical analyses were performed. We sampled the old leaf to determine changes of the defensive traits that were related to leaf maturation, whereas the sampling of the young and newly developing leaf was considered a proxy for effects related to plant age. Note that at growth stage L2 old and young leaves were identical, i.e. the second developed leaf.

For both experiments, teosinte seeds were first incubated on moistened filter paper (room temperature, no light) until germination (48–72 h). Once coleoptiles and roots had formed, seedlings were transferred to potting soil and grown as described above.

### Chemical analyses

Leaves were ground to a fine powder in liquid nitrogen. Depending on the growth stage between 20 and 50 mg of frozen plant powder were then suspended in 1 mL of acidified methanol/water (50:49.5, v/v; 0.5% formic acid) and five to eight glass beads were added. BX aglucones are most stable under acidic conditions [50, 51]. Furthermore, the use of acidified extraction solvent quenches any residual hydrolytic enzyme activity and thereby stabilises the glucosides and prevents the release of aglucones. Following agitation in a bead mill for 3 min (30 Hz) and centrifugation at 20,800 *g* for 5 min, 500 μL of supernatant were transferred to glass vials and stored at -80°C. BXs and maysin were analysed using a Waters Acquity UPLC system equipped with an Acquity BEH C18 column (2.1 x 50 mm, 1.7 μm particle size) that was connected to an eλ photodiode array (PDA) detector and a Synapt G2 QTOF mass spectrometer (Waters, Milford, MA, USA) in series as described in [52]. BX concentrations were calculated based on external calibration curves obtained from pure standards of DIMBOA-Glc, HDMBOA-Glc, and DIMBOA at 0.2, 1, 5 and 20 μg / mL or 0.2, 1, 2, 5, 10, 20, 40, 60 μg / mL, respectively. For highly concentrated plant extracts the signal intensities repeatedly exceeded the linear range of the MS detector, in which case the PDA detector (λ = 264 ± 1.2 nm) of the UPLC system was used for quantitation. Limits of quantitation (LOQs) for DIMBOA-Glc, HDMBOA-Glc and DIMBOA were below 3 μg / g FW. Presence of maysin in the plant extracts was confirmed by comparing retention time and mass spectrum to those of an authenticated standard. Maysin was quantified using the PDA detector at a wavelength of 350 nm (± 1.2 nm). Calibration curves were obtained from pure isoorientin 2”-*O*-rhamnoside at 0.2, 1, 2, 5, 10, 20, 50 and 100 μg / mL.

### Determination of leaf toughness

Leaf toughness was measured using a mechanical device consisting of a metal lever and a base plate, which were connected one-sidedly by a hinge [53] (S1 Doc). The lever was equipped with a little needle (Ø 1 mm) that fit through a whole (Ø 1.2 mm) in a counterpart on the base plate. A dynamometer (capacity = 100 g, division = 1 g, PESOLA AG, Baar, Switzerland) was attached on the end of the lever opposing the joint. Leaf toughness was measured as the force that was needed for the needle to pierce through the leaf. For the measurement, the leaf was placed on top of the base with the needle resting on top of the leaf. Then, the lever was pulled down manually by pulling down the dynamometer, while continuously reading the force that was applied. The force that caused the leaf to rupture was then recorded. For each leaf three measurements were taken at a 1.5 cm distance from each other in the middle of the leaf. At later growth stages, when leaf veins became clearly visible, all measurements were done in between the veins for reasons of comparability.

### Statistical analyses

Total BX levels were calculated as the sum of the seven major BX glucosides DHBOA-Glc, DIBOA-Glc, HMBOA-Glc, DIMBOA-Glc, DIM2BOA-Glc, HDMBOA-Glc and HDM2BOA-Glc. Statistically significant effects of growth stage and leaf position on foliar BX and maysin levels as well as leaf toughness were determined for each plant line separately by two-way analyses of variance (ANOVAs). Total BX levels in the first experiment were further analysed by three-way ANOVA in which domestication status, leaf type and growth stage were used as factors. In the second experiment BX levels were compared for each growth stage separately aiming to detect statistically significant effects of plant genotype and leaf position: for growth stage L2 a one-way ANOVA was carried out as only one leaf was sampled at this growth stage, whereas for growth stages L4 and L6 two-way ANOVAs were performed using plant genotype and leaf position as factors. All data were checked for normal distribution and homoscedasticity. If assumptions were not met, data were either log-transformed or ranked prior to ANOVA. When significant effects were indicated Holm-Sidak was used as *post-hoc* test. All statistical analyses were done in SigmaPlot for Windows, Version 12.5 (Systat Software, San Jose, CA, USA).

## Results

### Plant phenology and BX content

**Fig 1:**
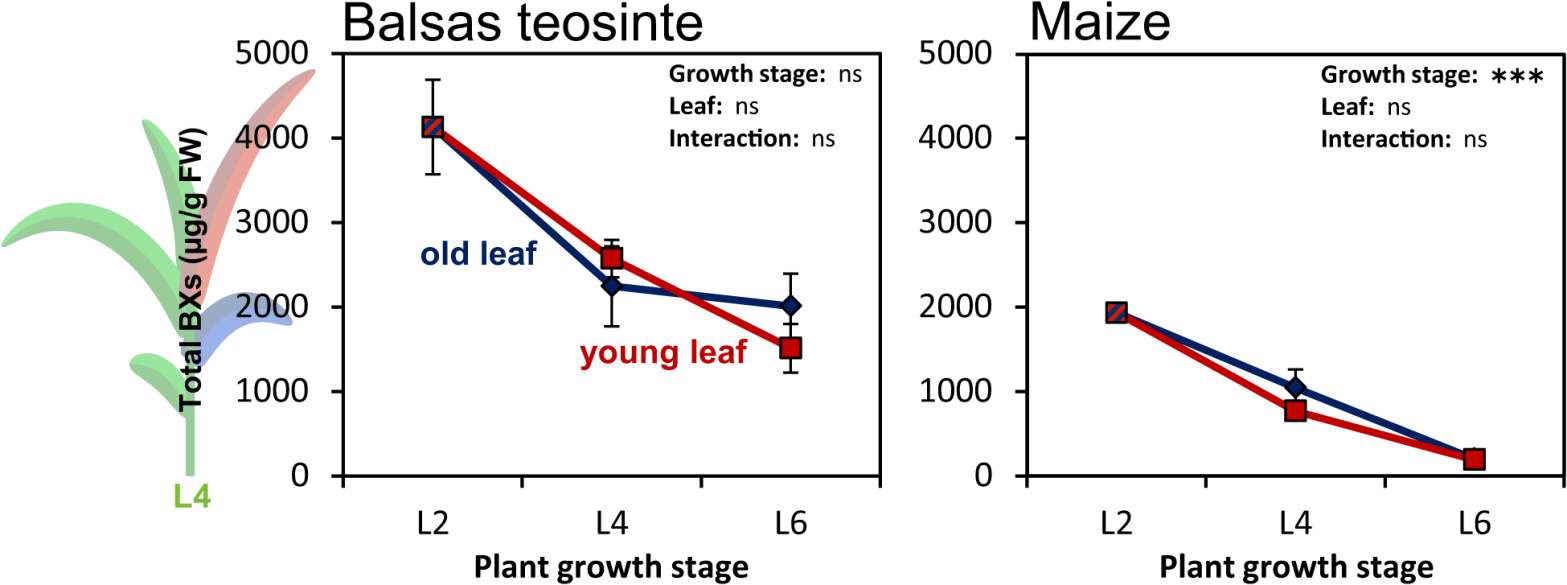
Pairwise comparison of constitutive BX levels in Balsas teosinte and maize. Total 1,4-benzoxazin-3-one (BX) concentrations were calculated as the sum of seven individual BX glucosides in one population of Balsas teosinte (left) and one maize inbred line (B73, right) at three different growth stages and are given as μg / g fresh weight (FW) (mean ± SE; N = 5–6). BX levels were monitored in old (blue) and young (red) leaves as indicated in the drawing to the left-hand side for growth stage L4. Note that at L2 old and young leaf were the same. Significant effects of growth stage and leaf identity on total BXs are indicated by asterisks: * P<0.05; ** P <0.01; *** P <0.001; ns: not significant; two-way ANOVA without growth stage L2. Chemical data for B73 has been published before [45].

In a first experiment we assayed the temporal dynamics of constitutive BX accumulation during early vegetative growth stages in the maize inbred line B73 and in the Balsas teosinte population Ejutla, for which the seeds were collected in its native habitat in Mexico [22]. As we were also interested in leaf-specific accumulation patterns, BXs were quantified in two different leaves (i.e. one old and one young leaf) at all three growth stages. In Ejutla total BX concentrations were highest at growth stage L2 (Fig 1) and showed slightly reduced levels at the subsequent growth stages. Plant age did not have a statistically significant effect on BX concentrations from growth stage L4 on (note that at growth stage L2 the old and the young leaf are the same, hence this growth stage was not considered in the two-way ANOVA). Furthermore, BX levels were very similar between the two different leaves at the last two growth stages. In the maize inbred line, BX levels were highest in young plants and declined sharply thereafter. No significant differences between different leaves within one growth stage were detected.

Compared to each other, Ejutla and B73 differed remarkably in their BX accumulation. Concentrations in Ejutla were at least twice as high as in B73 at the first two growth stages and even ten times higher at growth stage L6. Accordingly, the effect of domestication status on constitutive BX levels was highly significant (Table 1). Furthermore, the significant interaction between domestication status and growth stage reflected the contrasting rates of BX decline in the two plant lines.

**Table 1:** Three-way ANOVA table on constitutive BX levels in *Zea mays* ssp. *parviglumis* Ejutla and *Zea mays* ssp. *mays* cv. B73.

Factor:	F	Df	*P*
Dom:	**195.650**	**1**	**<0.001**
Leaf:	0.519	1	0.476
Stage:	**67.902**	**1**	**<0.001**
Dom * Leaf:	0.357	1	0.554
Dom * Stage:	**26.750**	**1**	**<0.001**
Leaf * Stage:	0.542	1	0.466
Dom * Leaf * Stage	2.417	1	0.128

A three-way ANOVA was performed, in which domestication status (Dom: Balsas teosinte or hybrid maize), leaf type (Leaf: old or young) and growth stage (Stage: L4 or L6) were included as factors. Statistically significant factors are highlighted in bold font type. All data were log10-transformed prior to ANOVA. Please note that growth stage L2 was excluded from the analysis as only one leaf was sampled.

Besides these quantitative differences, the two blends also differed qualitatively from each other. In teosinte, HDMBOA-Glc was the predominant compound, whereas DIMBOA-Glc was the most abundant BX in B73 (S1 Fig).

### Comparing BX levels in teosinte populations and cultivated maize lines

**Fig 2:**
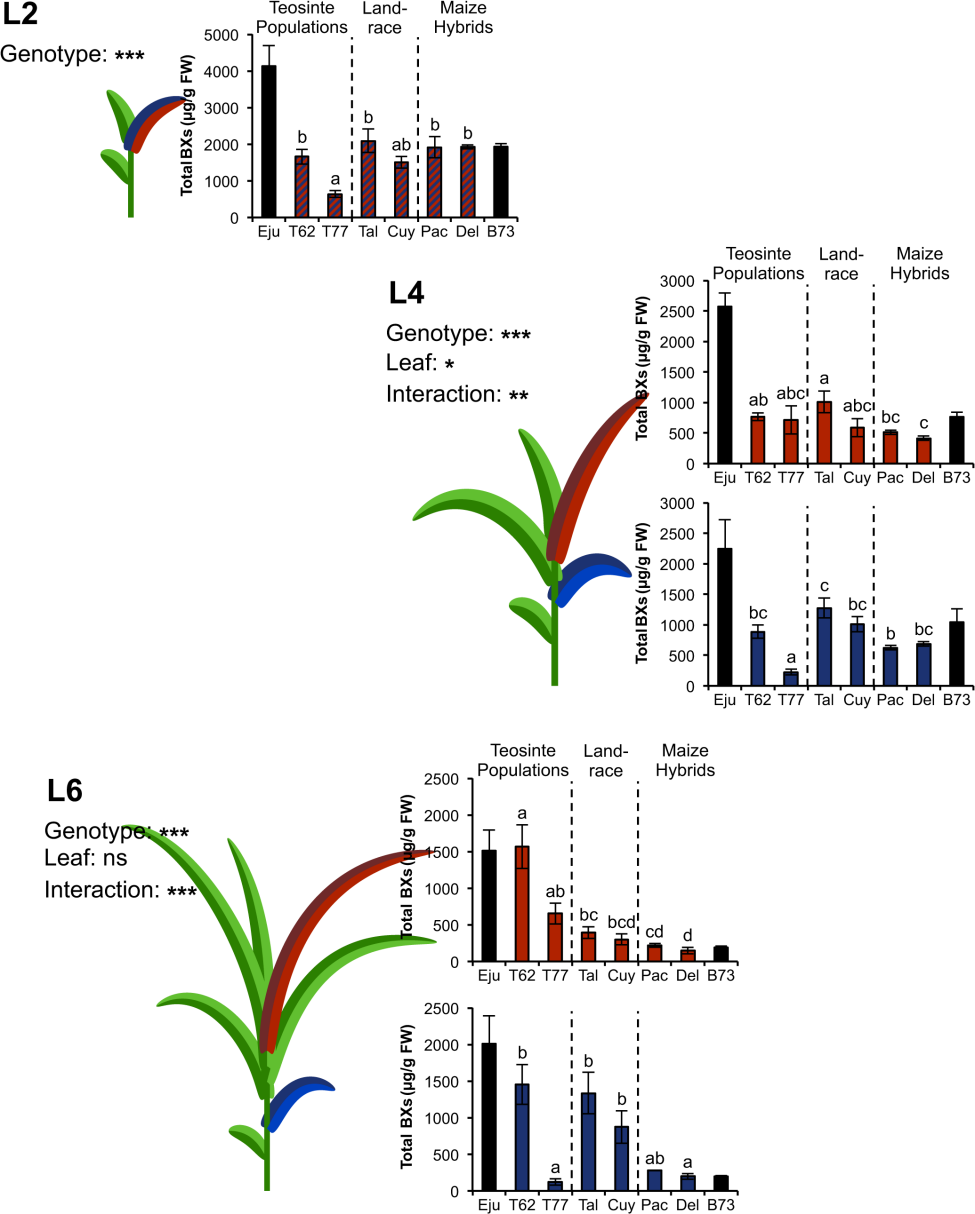
Constitutive BX levels in multiple representatives of teosinte and cultivated maize. Total levels of 1,4-benzoxazin-3-ones (BXs) at different growth stages of two teosinte accessions (T62 and T77), a Tuxpeño landrace variety from two collection sites (Talpitita and El Cuyotomate) and two maize hybrid lines (Pactol and Delprim) were calculated as the sum of seven individual BX glucosides and are given as μg / g fresh weight (FW) (mean ± SE; N = 3–5). At each growth stage old (blue) and young (red) leaves were sampled. Note that at L2 old and young leaf were the same. The specific positions of harvested leaves are highlighted in the maize drawings. Significant effects of plant genotype and leaf identity on total BXs are denoted by asterisks: * P<0.05; ** P <0.01; *** P <0.001; ns: not significant; two-way ANOVA without growth stage L2. Significant differences between plant lines are indicated by different letters and derive from Holm-Sidak tests for the respective leaf type. The concentrations of Ejutla and B73 from the previous figure are included for comparison (black bars).

The initial observations for Ejutla and B73 prompted us to investigate the effects of maize domestication on BX accumulation patterns during early ontogeny in more detail. Therefore, plants of two more teosinte accessions (T62 and T77), one Tuxpeño landrace collected from two different sites in Mexico (Talpitita and El Cuyotomate) and two modern maize varieties (Pactol and Delprim) were screened for their BX contents. To take into account possible differences between different teosinte taxa a population of another annual teosinte, *Z*. *mays* ssp. *mexicana* (T77), was included in addition to the Balsas teosinte accession T62. Hybridisation between maize and *Z*. *mays* ssp. *mexicana* occurs naturally where the two grow sympatrically [15, 16]. Surprisingly, we did not find as clear-cut accumulation patterns as in the pairwise comparison, nevertheless certain leaf-specific differences between the six lines were observed (Fig 2).

At the earliest growth stage, BX concentrations in the different lines were comparable to those observed for B73 in the first experiment and ranged from 1500 to 2100 μg / g FW with the exception of the teosinte accession T77. The latter contained the lowest amounts of constitutive BXs at approximately one third of the concentrations detected in the other lines.

At growth stage L4, a significant effect of leaf type on BX concentrations became apparent, in addition to strong genotype effects. However, a significant interaction between the two factors indicated that the effect of leaf type was genotype-dependent. For instance, for the maize landrace El Cuyotomate BX levels were higher in the old leaf, whereas for T77 those in the young leaf were higher. Overall, BX levels in the six additional lines were rather similar with the exception of the old leaf of T77, which contained much lower BXs than any other leaf or plant line.

At the last growth stage, the statistical analysis also revealed a significant interaction between leaf type and genotype, but more importantly within each leaf type highly significant differences between the different lines were observed. The old leaves of the teosinte accession T62 and of the two populations of the Tuxpeño landrace displayed approximately six times higher BX levels than those of T77, Pactol or Delprim. BX concentrations in the young leaves of Pactol and Delprim were comparable to those in the two landrace populations. The highest amounts were detected in the young leaves of T62.

When the temporal dynamics of BX accumulation were considered separately for each plant line, several patterns became evident. First, constitutive BX levels in the two teosinte populations changed relatively little over time (S2 and S3 Figs). In this respect no clear trend was observed for T62, but BX levels in T77 decreased specifically in the old, maturing leaf and remained rather constant in young, newly developing leaves leading to significantly higher concentrations in the latter as compared to the old leaf. Second, opposite trends were observed for the two Tuxpeño landrace populations. BX concentrations also declined sharply between L2 and L4. From growth stage L4 on, however, BX levels remained nearly constant in the old leaves whereas they decreased further in the young leaves (-50% from L4 to L6) resulting in three fold higher BX concentrations in old leaves as compared to young leaves (S4 and S5 Figs). Third and in contrast to the previous two observations, BX concentrations of the two maize lines diminished strongly over time irrespective of leaf type. For both leaves a decrease of around 90% was observed from L2 to L6 (S6 and S7 Figs). These observations were also reflected by a statistically significant interaction between domestication status and growth stage (linear mixed model, S1 Table). However, no significant effect of domestication status on constitutive BX levels was detected (S1 Table).

Regarding individual BX metabolites, most of them either remained at a constant level or decreased with plant age with the exception of DIBOA-Glc, whose concentrations increased significantly over time in the old, maturing leaf of all six plant lines (S2–S7 Figs).

### Foliar maysin levels increase with plant age in a leaf-specific manner irrespective of domestication status

**Fig 3:**
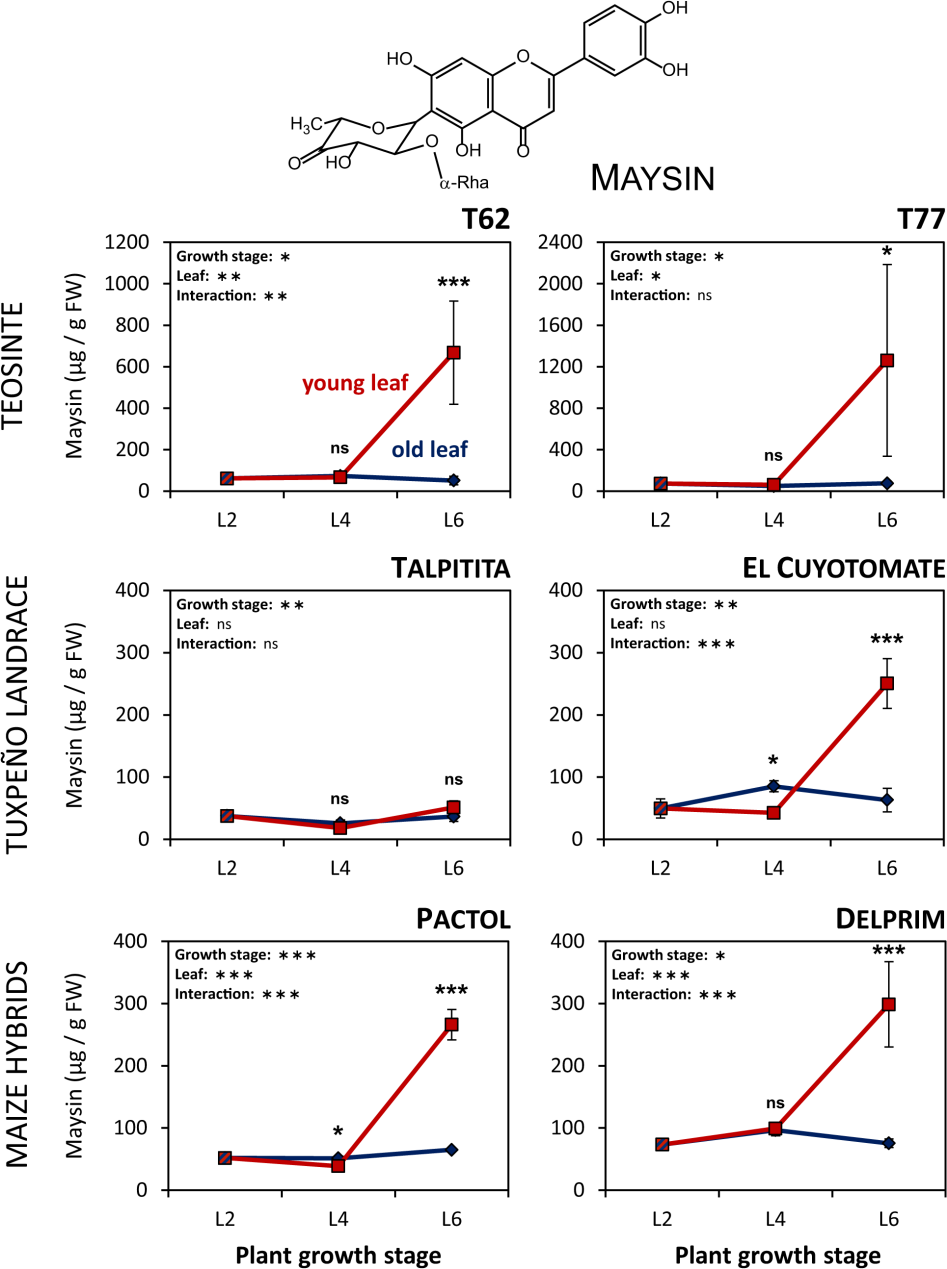
Maysin accumulates in newly developing leaves of maize and teosinte at later growth stages. Maysin concentrations were determined in old (blue) and young (red) leaves of two teosinte accessions (T62 and T77), a Tuxpeño landrace variety from two collection sites (Talpitita and El Cuyotomate) and two maize hybrid lines (Pactol and Delprim) at three early vegetative growth stages (L2, L4 and L6). Concentrations are given as μg / g fresh weight (FW) (mean ± SE; N = 3–5). Statistically significant effects of plant growth stage or leaf identity are denoted by asterisks (*P < 0.05; **P < 0.01; ***P < 0.001; ns: not significant; two-way ANOVA without growth stage L2).

In parallel to BXs, the glycosylated flavonoid maysin was quantified in the same plant parts and an opposite accumulation pattern was observed (Fig 3). While maysin concentrations did hardly change in old leaves across all three growth stages and remained below 100 μg / g FW, a significant increase was observed in young leaves between L4 and L6. This increase was evident for all plant lines examined except in the Tuxpeño landrace, whose seeds were collected in Talpitita. The two teosinte accessions displayed the highest maysin concentrations that were measured with 1260 μg / g FW in young leaves of T77 and 668 μg / g FW in young leaves of T62, respectively. However, even though maysin concentrations in the young leaves at growth stage L6 were affected by plant genotype (one-way ANOVA, F_5,22_ = 7.296, *P* < 0.001), maysin concentrations between the two teosinte accessions, the two maize hybrids Pactol and Delprim and the Tuxpeño landrace from El Cuyotomate did not differ significantly from each other (*post-hoc*: Holm-Sidak test). Accordingly, the overall effect of domestication on maysin content was not statistically significant (linear mixed model, S2 Table). The two maysin derivatives apimaysin and 3”-methoxymaysin were not present in any of the plant lines.

### Leaf toughness is not affected by domestication

**Fig 4:**
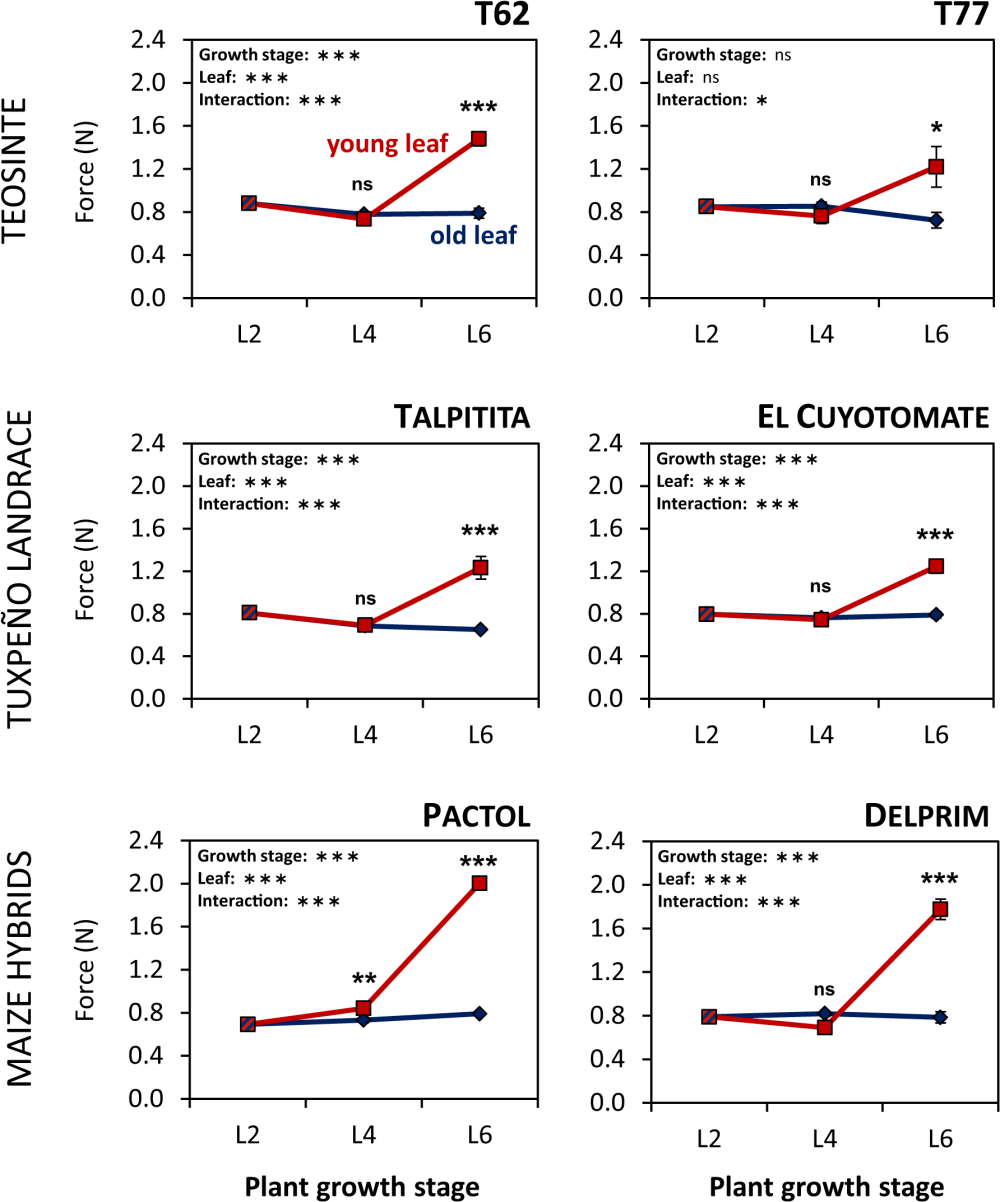
Leaf toughness in maize and teosinte increases in a leaf-specific manner during early ontogeny. Leaf toughness was measured in old (blue) and young (red) leaves of two teosinte accessions (T62 and T77), a Tuxpeño landrace variety from two collection sites (Talpitita and El Cuyotomate) and two maize hybrid lines (Pactol and Delprim) at three early vegetative growth stages (L2, L4 and L6). Leaf toughness is expressed as the force needed for penetration of the leaf blade and given in Newton (N) (mean ± SE; N = 3–5). Statistically significant effects of plant growth stage or leaf identity on leaf toughness are denoted by asterisks (*P < 0.05; **P < 0.01; ***P < 0.001, ns: not significant; two-way ANOVA without growth stage L2).

The force needed to penetrate the leaf blade was measured as an indicator of leaf toughness. Interestingly, leaf toughness followed a temporal pattern that was similar to the one of maysin accumulation. Hardness of old leaves did not change during early vegetative growth (Fig 4). The force needed to penetrate these leaves remained unaffected at approximately 0.8 N across all three growth stages and in all plant lines. In young and newly developing leaves, however, leaf toughness did increase considerably between L4 and L6. Whereas during the first two growth stages the force necessary for leaf rupture of young leaves was nearly identical to the force that was needed for old leaves, values were approximately two-fold higher at the last growth stage leading to statistically significant differences between newly developing and older leaves in all six plant genotypes. Accordingly, significant interaction terms between the two factors further supported that the observed effect of plant age was leaf-specific. Furthermore, growth stage and leaf position had very significant effects on leaf toughness in five out of six plant lines, the exception being the teosinte accession T77. Most strikingly, newly developing leaves of the two maize hybrid lines Pactol and Delprim displayed the highest leaf toughness values at growth stage L6 (S8 Fig). Overall, however, the effect of domestication on leaf toughness was not statistically significant (linear mixed model, S3 Table), although a significant interaction between domestication status, leaf type and growth stage was detected (S3 Table) suggesting leaf- and growth stage-specific effects of domestication.

## Discussion

In a recent meta-analysis on the ontogeny of plant defence and herbivory including 116 scientific publications, Koricheva & Barton [41] revealed certain ontogenetic patterns. However, these patterns were only valid within smaller subgroups, e.g. within plants belonging to the same life form (i.e. woody, herbaceous or grass), while no general ontogenetic trajectories became evident. Concerning grasses in particular, the authors recognised a lack of data on the ontogenetic trajectories of the various defensive compounds. This lack, in combination with the assumption that most modern crop varieties are less resistant to attack by insect herbivores compared to their wild ancestors [3], prompted us to carry out an extensive analysis of leaf toughness and constitutive accumulation of two classes of defensive secondary metabolites during early vegetative growth in different teosinte populations and maize cultivars. In recent years, evidence accumulated indicating a loss of resistance in the teosinte/maize system due to domestication. The underlying mechanisms, though, remained for the most part elusive. In one case enhanced performance of the well-adapted maize-feeder *S*. *frugiperda* on cultivated maize was found to be related to decreased transcript levels of certain protease inhibitors [20], while in another case preferential oviposition of the specialist leafhopper *D*. *maidis* on maize coincided with decreasing leaf toughness along the domestication gradient [21].

### Plant ontogeny exerts contrasting effects on different defence traits in maize and teosinte

BXs represent the predominant class of secondary defence metabolites in maize and are toxic to a broad range of insect herbivores [24]. Their presence has been reported for different teosinte taxa: leaf levels of DIMBOA-Glc were found to be almost identical in a comparison between a maize cultivar, a Balsas teosinte and another wild relative of maize, *Z*. *mays* ssp. *mexicana* [54]. Intriguingly, for the latter it has been reported that the roots contain four times lower BX levels than those of cultivated maize [34]. Nonetheless, based on the various studies that report better herbivore resistance in the wild ancestor we hypothesised that teosinte accumulates higher amounts of BXs than cultivated maize. In contrast to this expectation we did not find such a pattern. In fact, the *Z*. *mays* ssp. *mexicana* population T77 even displayed the lowest BX concentrations in the old leaf at all three growth stages. We further found that the differences in total BX levels between the remaining Balsas teosinte population T62, the two Tuxpeño landrace populations and the two maize hybrids Pactol and Delprim were rather modest at the first two growth stages. However, we observed substantial quantitative differences among the various genotypes for the last growth stage. These differences were mainly due to strong differences in the rates by which the total BXs decreased. This decrease over the three plant stages was more evident for the cultivated maize lines than for the landraces and teosintes (S2–S7 Figs). BX concentrations in the two maize hybrid lines underwent a steep decline of about 90% from L2 to L6, irrespective of leaf position. This suggests that in maize, in successively developing young leaves, less substrate is converted into BXs, while a different mechanism has to be responsible for the strong decline of BX levels in maturing, old leaves. Overall, our data suggest that other factors than BXs mediate the divergent resistance phenotypes between maize and teosinte. However, it has to be noted that most of the cited studies on herbivore resistance in the teosinte/maize system examined plants at later growth stages (i.e. L4 or later). Thus, a contribution of more pronounced differences in BX concentrations at later growth stages to the observed resistance pattern cannot be entirely excluded.

Contrary to our expectations, maysin concentrations increased with plant-age in a leaf-specific manner. Maysin contents in newly developing leaves of teosinte tended to be higher than those in leaves of the maize cultivars, but these differences were not statistically significant, probably due to large variability among the teosinte samples. Maysin is a potent defence metabolite, which slows down the growth of *H*. *zea* as well as of *S*. *frugiperda* caterpillars [35, 36, 55]. Fall armyworm larvae that were forced to feed on artificial diet containing maysin at a concentration of 1.5 mM gained approximately 40% less weight compared to non-exposed conspecifics [36]. Concentrations beyond 2.5 mM even hampered their growth by more than 60%. The highest maysin levels that were found for the two teosinte accessions corresponded to concentrations of 1.3 and 2.4 mM for T62 and T77, respectively. Hence, these concentrations are well within a range that causes serious growth reductions in *S*. *frugiperda*. Although concentrations in the maize cultivars were considerably lower than those in teosinte, it is tempting to hypothesise that maysin still provides an effective defence against leaf-feeding herbivores in maize leaves during later vegetative growth stages. Yet, further investigations into the ontogenetic trajectory of this metabolite are needed to determine whether the observed increase in concentration is persistent.

Concerning the toughness of maize and teosinte leaves we detected considerable variation across the three growth stages. While leaf maturation did not have any effect on this trait, newly developed, young leaves were tougher than their older predecessors resulting in leaf-specific effects of plant age on leaf hardness. This observation was in good agreement with our hypothesis that leaf toughness increases with plant age but remains constant during leaf aging. In maize, this physical defence trait is negatively correlated with performance of the two corn borers *O*. *nubilalis* and *Diatraea grandiosella*, as well as leaf-feeding *S*. *frugiperda* caterpillars [46, 56]. Williams et al. [46] found that the leaves of resistant maize cultivars, which experienced less damage from fall armyworm and southwestern corn borer infestation, were tougher than those of susceptible cultivars. Furthermore, they observed that the hardness of maize leaves increased with plant age. In that study, unfortunately, leaf toughness was only measured in the two youngest whorl leaves using a comparable leaf penetration method. Taken together with our observations, the data suggest that within an individual maize plant leaf toughness increases from bottom to top. In turn, this pattern may influence the foraging behaviour of leaf-feeding herbivores that colonise their host plant from the soil. By all means, from the plant’s perspective adaptive values other than defence may well have contributed to shaping the observed ontogenetic pattern. For instance, it seems intuitive that larger and heavier leaves, as during later growth stages, simply require a more solid structure to support themselves. It can be expected that this need for rigidity will be even more evident for the later plant stages, especially in the tall and massive cultivated plants. Indeed, the two maize hybrid lines showed the highest leaf toughness values. This is in contrast to earlier studies that reported a decrease of leaf toughness from Balsas teosinte to maize [21]. However, in that study only the force that was needed to penetrate the midrib was of interest as its relationship to the oviposition preferences of a specialist leafhopper were examined. Thus, whether these contrasting leaf toughness patterns do exist or whether they derive from differences in measuring criteria remains unclear.

### Pairwise comparisons may lead to wrong conclusions on effects of domestication

Recently, Turcotte et al. [4] investigated the effect of 29 independent domestication events on the performance of the generalist herbivores *Spodoptera exigua* (caterpillar) and *Myzus persicae* (aphid). The authors came to the conclusion that, in general, domestication did reduce plant resistance to *S*. *exigua* (but not to *M*. *persicae*) but the effect varied considerably for different wild relative/crop systems. Interestingly, survival and growth of *S*. *exigua* larvae did not differ between teosinte and maize in that study [4]. Those findings contradict the ones mentioned earlier, which reported significant domestication effects on herbivore resistance in maize [18]. While one may argue that different plant genotypes or herbivore species (*S*. *frugiperda* vs. *S*. *exigua*) were used in the different studies it would be expected that the effect would be stronger for the generalist (*S*. *exigua*), as was found in other plant-herbivore systems [57]. An alternative explanation for the discrepancies between the results from different studies is that most of them only used pairwise comparisons between one teosinte accession and one maize cultivar. In our first experiment we also used teosinte plants originating from only one natural population and compared their constitutive BX levels to those of a single maize inbred line. Based on this pairwise comparison one would have clearly concluded that Balsas teosinte does contain significantly higher BX levels than cultivated maize (Fig 1). By expanding the range of plant genotypes in our analysis great variability among the teosintes has been revealed.

We argue that similarly variable outcomes can be expected for other traits, such as herbivore performance. This may apply in particular to wild relative/crop systems where a high genetic diversity has been maintained throughout the domestication bottleneck as is the case for maize [14]. Further support for this consideration is provided by a comparison of volatile emissions from teosintes belonging to different taxa and 11 maize cultivars [47]. Maize plants, as is the case for many plant taxa, release volatile organic compounds (VOCs) following herbivore-feeding [58, 59]. These VOCs can serve as alert signals for undamaged leaves [60–65] and as cues for herbivore enemies to locate their prey or host insects [66–68]. In a previous comparison among eleven maize cultivars and five teosinte taxa volatile emissions were found to vary greatly in terms of quantity, but qualitative differences were minimal [47]. All major compounds that were emitted by the different teosintes upon induction treatment were also detected in the headspace of induced maize plants. These observations were interpreted as a result of rather small bottleneck effects during on the genetic diversity in maize.

It is noteworthy though, that cultivated maize varieties of distinct geographic origins can be distinguished based on at least one polymorphism in their VOC blends: most maize varieties derived from North American breeding programmes have lost their ability to produce β-caryophyllene [69] an important foraging cue for soil borne entomopathogenic nematodes [49, 70]. This loss could be merely a by-product of genetic drift, but might also be the result of unwitting artificial selection, as β-caryophyllene is also attractive to pest insects [71–73].

The relative composition of BXs also varies with the geographic origin of maize lines. Inbred lines derived from tropical germplasm consistently show higher HDMBOA-Glc /DIMBOA-Glc ratios [52]. In modern cultivars from more temperate regions there is a bias towards DIMBOA-Glc that could be the result of artificial selection for resistance against aphids. Various leaf-chewers are able to reglucosylate and thus efficiently detoxify DIMBOA, but no detoxification route for HDMBOA is known, making it a more effective defence metabolite against chewers [30–32]. DIMBOA, on the other hand, mediates resistance against aphids *via* induction of callose deposition. Indeed, higher DIMBOA-Glc concentrations in maize cultivars from temperate zones have been associated with a latitudinal shift in insect pest pressure towards piercing-sucking herbivores [28, 52]. The highly bred maize cultivars that were used in the present study all originate from breeding programs in temperate regions. In agreement with the previous findings [28, 52] DIMBOA-Glc was the predominant BX at all growth stages in these lines. As expected, the BX blends of the two Balsas teosinte accessions Ejutla and T62 were dominated by HDMBOA-Glc. However, the *Z*. *mays* ssp. *mexicana* accession T77 showed an intermediate BX composition with a more balanced HDMBOA-Glc/DIMBOA-Glc ratio. The Mexican landrace populations also showed a ratio that was slightly biased towards HDMBOA-Glc.

### Concluding remarks

In contrast to the prevailing notion that cultivated crop plants have reduced defensive capacities as compared to their wild relatives, we did not observe higher defence levels in teosinte for the measured traits. Differences in constitutive BX levels failed to sufficiently explain why close relatives of maize are frequently reported to be more resistant to herbivorous insects than cultivated maize varieties. Yet, there was a striking difference in the decrease of BX levels with age, which was far more evident for the cultivated maize lines than for the landraces and teosintes. For the two other defence traits maize and two ancestral teosinte taxa showed similar ontogenetic trajectories. In all plant lines, leaf toughness and maysin concentrations increased in newly developing leaves but remained constantly low in old, maturing leaves during early vegetative growth indicating leaf-specific effects of plant age on those two traits. We thus conclude that, most likely, other defence traits contribute to the distinct resistance phenotypes.

Furthermore, we demonstrate that mere pairwise comparisons may lead to false conclusions regarding the effects of domestication on defensive (and possibly other) traits in the teosinte/maize system. Instead, replicated comparisons with multiple genotypes are needed for conclusive evaluations of the domestication effects. Our study shows that the generally assumed reduction in defence compounds in crops may not always apply. Further studies with appropriate replication are needed to determine how general these patterns are.

## Supporting Information

S1 Doc Description of mechanical device for leaf toughness measurements. (DOCX)

S1 Table Linear Mixed Model table for foliar BX concentrations. (DOCX)

S2 Table Linear Mixed Model table for foliar maysin concentrations. (DOCX)

S3 Table Linear Mixed Model table for leaf toughness. (DOCX)

S1 Fig Relative composition of BX blends in *Z*. *mays* ssp. *parviglumis* Ejutla and *Z*. *mays* ssp. *mays* var. B73. The relative BX composition of each leaf at the different growth was calculated based on the concentrations that were measured for the seven BX glucosides DHBOA-Glc, DIBOA-Glc, HMBOA-Glc, DIMBOA-Glc, DIM2BOA-Glc, HDMBOA-Glc and HDM2BOA-Glc.
(EPS)

S2 Fig Concentrations of individual BXs in *Z*. *mays* ssp. *parviglumis* accession T62. Concentrations of individual metabolites in old (blue) and young (red) leaves during early growth stages are given as μg / g FW (mean ± SE; N = 3–5). Significant effects of growth stage or leaf identity were determined by two-way ANOVA and denoted by asterisks: * P<0.05; **P<0.01; ***P<0.001; ns: not significant. LOD: limit of detection.
(EPS)

S3 Fig Concentrations of individual BXs in *Z*. *mays* ssp. *mexicana* accession T77. Concentrations of individual metabolites in old (blue) and young (red) leaves during early growth stages are given as μg / g FW (mean ± SE; N = 3–5). Significant effects of growth stage or leaf identity were determined by two-way ANOVA and denoted by asterisks: * P<0.05; **P<0.01; ***P<0.001; ns: not significant. LOD: limit of detection.
(EPS)

S4 Fig Concentrations of individual BXs in a Tuxpeño landrace of *Z*. *mays* ssp. *mays* collected in Villa Purificaciòn municipality, Mexico (Talpitita). Concentrations of individual metabolites in old (blue) and young (red) leaves during early growth stages are given as μg / g FW (mean ± SE; N = 5). Significant effects of growth stage or leaf identity were determined by two-way ANOVA and denoted by asterisks: * P<0.05; **P<0.01; ***P<0.001; ns: not significant. LOD: limit of detection.
(EPS)

S5 Fig Concentrations of individual BXs in a Tuxpeño landrace of *Z*. *mays* ssp. *mays* collected in Ejutly municipality, Mexico (El Cuyotomate). Concentrations of individual metabolites in old (blue) and young (red) leaves during early growth stages are given as μg / g FW (mean ± SE; N = 5). Significant effects of growth stage or leaf identity were determined by two-way ANOVA and denoted by asterisks: * P<0.05; **P<0.01; ***P<0.001; ns: not significant. LOD: limit of detection.
(EPS)

S6 Fig Concentrations of individual BXs in *Z*. *mays* ssp. *mays* var. Pactol. Concentrations of individual metabolites in old (blue) and young (red) leaves during early growth stages are given as μg / g FW (mean ± SE; N = 5; except old leaf at L6: N = 1). Significant effects of growth stage or leaf identity were determined by two-way ANOVA and denoted by asterisks: * P<0.5; **P<0.01; ***P<0.001; ns: not significant. LOD: limit of detection.
(EPS)

S7 Fig Concentrations of individual BXs in *Z*. *mays* ssp. *mays* var. Delprim. Concentrations of individual metabolites in old (blue) and young (red) leaves during early growth stages are given as μg / g FW (mean ± SE; N = 4–5). Significant effects of growth stage or leaf identity were determined by two-way ANOVA and denoted by asterisks: * P<0.05; **P<0.01; ***P<0.001; ns: not significant. LOD: limit of detection.
(EPS)

S8 Fig Effects of plant genotype and leaf identity on leaf toughness. Leaf toughness was measured in old (blue) and young (red) leaves of teosinte and maize plants at three early vegetative growth stages (L2, L4 and L6). Leaf toughness is expressed as the force needed for penetration of the leaf blade and given in Newton (N) (mean ± SE; N = 3–5). Statistically significant effects of plant genotype or leaf identity on leaf toughness were determined by one-way or two-way analyses of variance, respectively. Analyses were performed on ranked data when necessary. For growth stage L2 Tukey was used as *post-hoc* test whereas Holm-Sidak was used as *post-hoc* test for growth stages L4 and L6. Statistically significant effects are denoted by asterisks (*P < 0.05; **P < 0.01; ***P < 0.001, ns: not significant). Different letters on top of the bars indicate statistically significant differences.
(EPS)
