# Supplementary material for: Maize Domestication and Anti-Herbivore Defences: Leaf-Specific Dynamics during Early Ontogeny of Maize and Its Wild Ancestors
Source: PLoS One. 2015 Aug 12;10(8):e0135722. doi: 10.1371/journal.pone.0135722 (PMC4534137; doi:10.1371/journal.pone.0135722)
Supplement: S1 Doc — (DOCX) [file pone.0135722.s001.docx]

S1. Device for leaf toughness measurements

Leaf toughness was measured using a mechanical device that was built by the mechanics’ workshop at the University of Neuchâtel. Basically, this device consisted of a lever that was fixed to a basal plate on one side and had a dynamometer attached to the other side. A little needle was fixed to the centre of the lever. On the basal plate, this needle was able to penetrate the hole of a miniature metal table. A counter weight was fixed to one end of the lever to balance its own weight.


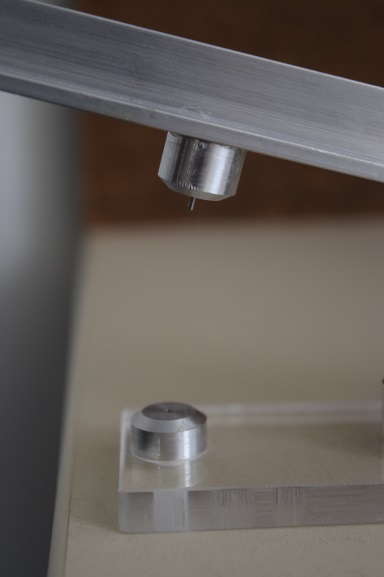

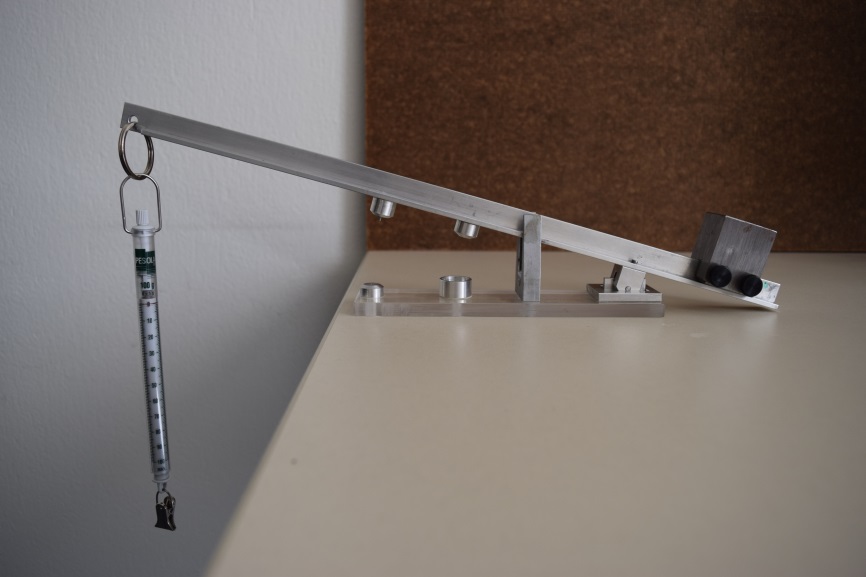


**counter weight**

**lever**

**basal plate**

**needle**

**table**

**dynamometer**

**needle**

**table**

To measure its toughness, a maize leaf was laid on top of this table and the lever was placed carefully right on top of the leaf. Then, the dynamometer was slowly pulled downwards until the leaf blade ruptured, constantly reading the applied force, which was given in [g] (see below).


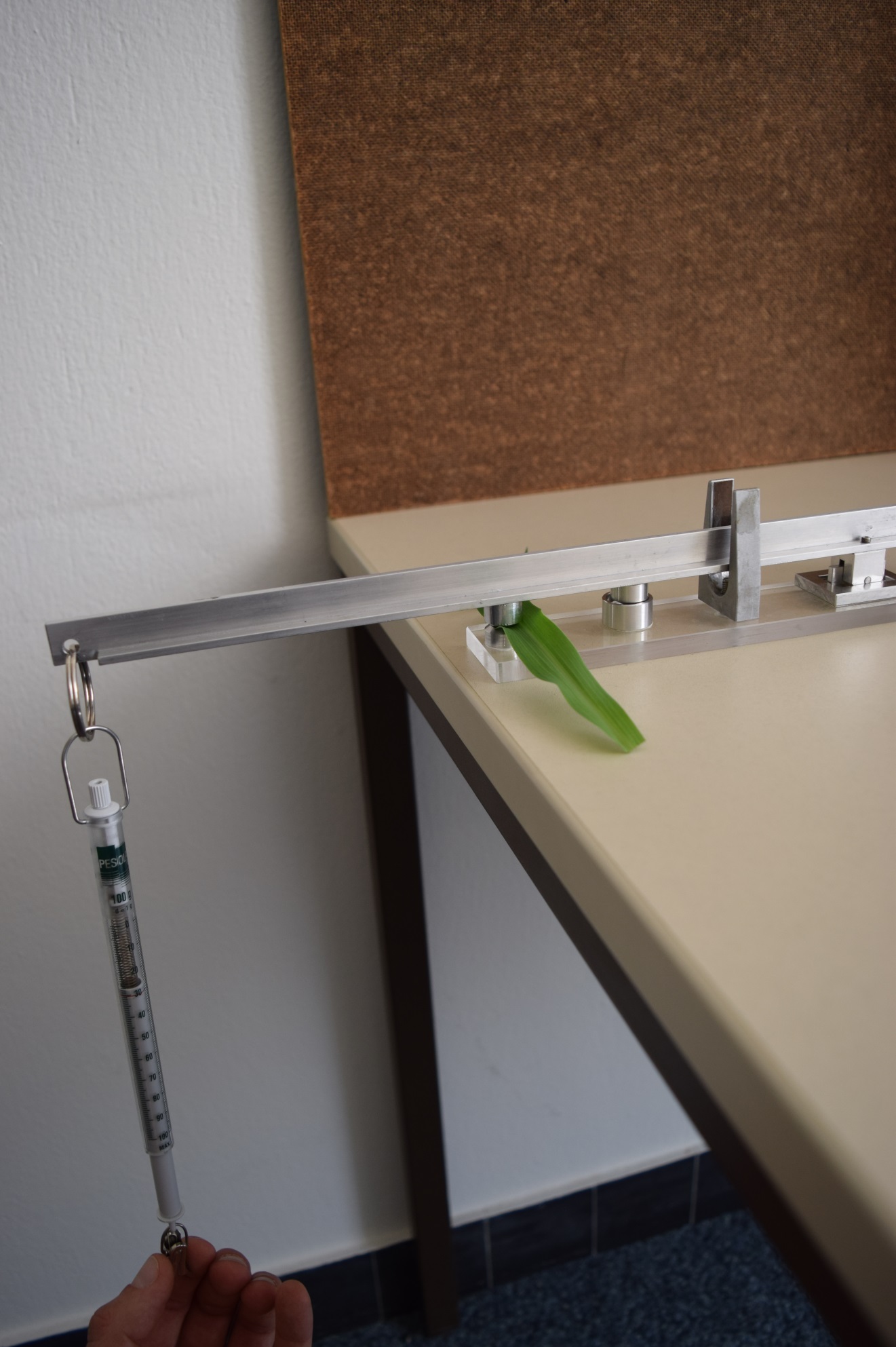


**F_2_**

**F_1_**

**l_2_ = 15 cm**

**l_1_ = 30 cm**

The applied force, given in [g], was then converted to [N] using the following formula:

F [N] = m [kg] * g with g = 9.81 N/kg

According to the following formula the force needed for leaf rupture (F_2_) was calculated as:

F_2_ [N] = F_1_ [N] * l_1_/l_2_ with l_1_ = 2l_2_, hence: F_2_ [N] = F_1_ [N] * 2
