# Supplementary material for: Maize Domestication and Anti-Herbivore Defences: Leaf-Specific Dynamics during Early Ontogeny of Maize and Its Wild Ancestors
Source: PLoS One. 2015 Aug 12;10(8):e0135722. doi: 10.1371/journal.pone.0135722 (PMC4534137; doi:10.1371/journal.pone.0135722)
Supplement: S1 Table — (DOCX) [file pone.0135722.s002.docx]

S1 Table. Linear Mixed Model table for foliar BX concentrations in two teosinte accessions (T62 and T77), a Tuxpeño landrace maize (Tal and Cuy) and two modern maize hybrid lines (Pac and Del). Statistically significant factors are indicated in bold font type. Dom: domestication status (teosinte, Tuxpeño landrace or hybrid maize); Leaf: leaf type (old or young); Stage: growth stage (L4 or L6).

| Factor: | Chisq | Df | pr(>Chisq) |
| --- | --- | --- | --- |
| Dom: | 0.6065 | 2 | 0.7384 |
| Leaf: | **5.1538** | **1** | **0.0232** |
| Stage: | **24.2459** | **1** | **<0.0001** |
| Dom * Leaf: | **39.4110** | **2** | **<0.0001** |
| Dom * Stage: | **26.4924** | **2** | **<0.0001** |
| Leaf * Stage: | 0.2359 | 1 | 0.6272 |
| Dom * Leaf * Stage | **6.9402** | **2** | **0.0311** |

The statistical analysis was performed using the lme4 package ([Bates et al., 2014](#_ENREF_1)) in R, version 3.1.3 (<http://www.R-project.org/>). Data was analysed using a Wald test on a linear mixed model (function “lmer”) with the fixed factors “domestication status”, “leaf type” and “growth stage”. Plant line was considered a random factor. BX concentrations were log10-transformed prior to analysis. Growth stage L2 was excluded from the analysis as only one leaf was sampled.

Bates D, Maechler M, Bolker B & Walker S (2014) lme4: Linear mixed-effects models using Eigen and S4. R package version 1.1–7. URL <http://CRAN.R-project.org/package=lme4>.
